# Delay Correction Method Based on VLF Timing Signal Phase Variation Model

**DOI:** 10.3390/s26113295

**Published:** 2026-05-22

**Authors:** Xinze Ma, Wenhe Yan, Zhaopeng Hu, Jiangbin Yuan, Yu Hua, Shifeng Li

**Affiliations:** 1 National Time Service Center, Chinese Academy of Sciences, Xi’an 710600, China; maxinze21@mails.ucas.ac.cn (X.M.); ywh@ntsc.ac.cn (W.Y.); huzhaopeng@ntsc.ac.cn (Z.H.); yuanjiangbin@ntsc.ac.cn (J.Y.); hy@ntsc.ac.cn (Y.H.); 2 School of Electronics, Electrical and Communications Engineering, University of Chinese Academy of Sciences, Xi’an 710600, China

**Keywords:** integrated PNT system, very-low-frequency, waveguide mode propagation, propagation-delay correction

## Abstract

**Highlights:**

**What are the main findings?**

- A phase-variation-based propagation-delay correction method is developed for very-low-frequency (VLF) timing signals.
- The corrected standard deviation is reduced to 2.0054–2.2500 μs for the Chongqing paths and 2.7987–4.4792 μs for the Guilin paths, while the corrected RMSE ranges from 2.1316 μs to 4.5641 μs across the six Alpha propagation paths.

**What are the implications of the main findings?**

- The results show that the proposed method can effectively suppress the dominant periodic propagation-delay variation in historical Alpha/VLF timing observations.
- The method provides a feasible model-based correction route for VLF receiver-side timing applications when external real-time correction information is unavailable.

**Abstract:**

Positioning, navigation, and timing (PNT) services require stable time transfer, but satellite-based PNT signals are vulnerable to interference, attenuation, and limited availability in constrained environments. Very-low-frequency (VLF) signals propagate over long distances in the Earth–ionosphere waveguide and can serve as a terrestrial complement to satellite-based timing systems. Their timing performance, however, is affected by propagation-delay variation, especially the diurnal component associated with changes in the effective ionospheric reflection height. This study presents a propagation-delay correction method for VLF timing signals based on a phase-variation model. The total delay error is separated into primary path delay, secondary propagation delay, and residual random error. The primary delay is calculated from the transmitter–receiver path, while the periodic secondary delay is corrected using the predicted phase variation. Historical Alpha observations recorded at Chongqing and Guilin were used to evaluate the correction performance. The results show that the corrected standard deviation is reduced to 2.0054–2.2500 μs for the Chongqing paths and 2.7987–4.4792 μs for the Guilin paths. The corrected root mean square error (RMSE) ranges from 2.1316 μs to 4.5641 μs across the six Alpha propagation paths. These results indicate that the proposed method can suppress the main diurnal propagation-delay component in the selected historical Alpha datasets, although further validation with contemporary and multi-season VLF observations is still needed.

## 1. Introduction

PNT refers to positioning, navigation, and timing. Location and time information are commonly obtained through radio navigation. As modern systems become more dependent on reliable position and time references, the role of PNT has become increasingly prominent [1,2,3]. Current timing architectures can be broadly grouped into space-based (satellite) and terrestrial systems, with the latter mainly implemented via longwave and shortwave transmissions [1,2,3]. Satellite navigation offers global coverage and high accuracy, but its received signal is weak and therefore vulnerable to interference; it is also difficult to use in underwater or underground environments [1,2,3]. Shortwave timing supports long-range transmission and relatively simple reception, but it typically provides poorer accuracy and continuity and is likewise impractical underwater or underground. Longwave timing is characterized by phase stability and comparatively strong interference tolerance, but its coverage and penetration depth are limited [2,4].

Space-based radio navigation can provide high-precision positioning and timing across the Earth’s surface and near-Earth space, yet the low received power makes it particularly sensitive to external interference [1,2,3]. In contrast, terrestrial radio navigation signals are stronger and generally more robust, and thus can serve as a backup to satellite-based systems [1,2]. Nevertheless, existing synchronization approaches still face constraints, including limited capability for ultra-long-distance synchronization and the practical infeasibility of underwater radio timing given the propagation behavior of higher-frequency signals [2,3,4]. VLF signals operate in the 3–30 kHz band, corresponding to wavelengths of approximately 100–10 km [5,6,7,8]. VLF propagation can exhibit relatively small path attenuation under favorable Earth–ionosphere waveguide conditions, with reported values on the order of 2–3 dB per 1000 km in the literature, although the actual attenuation depends on frequency, propagation path, ground parameters, and ionospheric conditions [5,6,7,8,9]. In addition, VLF signals can penetrate seawater to depths of roughly 10–40 m (30–130 ft), which enables underwater communication [5,7,9]. These properties make VLF a useful complement to terrestrial timing and synchronization systems [4,5,6,7,8].

With the exception of the five Russian Alpha stations, which use continuous-wave (CW) modulation, most VLF transmitting stations employ minimum shift keying (MSK) [9,10,11]. Because VLF signals can penetrate certain obstacles (e.g., rock and building materials) more effectively than higher-frequency signals and can propagate over long distances, they have been used in military and sensing-related applications [5,7,9]. VLF station transmissions are generally stable; analyses of their propagation paths and of temporal variations in received signals can be used to infer changes in the ionosphere [5,12,13,14]. VLF receivers typically monitor both station transmissions and atmospheric signals, and they have been applied in solar-activity monitoring, ground-based geospatial remote sensing, and maritime communication and navigation systems [12,13,14]. VLF timing signals are generated from highly stable time and frequency references, yielding favorable signal properties for timing. Beyond providing timing services, these signals also support studies in geospace environment monitoring, solar-activity monitoring, and communications, and they contribute to the development of related foundational techniques [4,12,13,14,15,16].

To date, relatively few navigation and timing systems have been built around VLF. The United States previously deployed the Omega navigation system, but it was later discontinued; at present, only a limited number of stations appear to be conducting low-frequency correlation experiments. Within the recent U.S. STOIC program, one research direction is the development of an ultra-long-range radio navigation and timing system based on VLF. Its navigation testing has been completed, and a positioning accuracy of 40 m has been reported after time-delay correction [1,11]. Russia’s Alpha navigation system, which was previously operational, is currently out of service for reasons that have not been specified [5,10,11,12,17,18]. Recent studies also indicate that VLF signals remain valuable for navigation-related and propagation-related research. For example, Yan et al. proposed a multichannel orthogonal correlation method for Alpha navigation signal detection under noise and mutual interference, showing that Alpha remains a useful signal source for VLF navigation, timing, and ionospheric studies [18]. In the broader positioning, navigation, and timing (PNT) field, Yang et al. discussed ground-based low-frequency and very-low-frequency longwave radio stations as part of comprehensive PNT infrastructures, indicating the potential role of LF/VLF signals in resilient PNT systems [19]. In addition, Sakaya and Takahashi investigated a VLF electromagnetic undersea positioning system using a receiving antenna array, further reflecting the potential of VLF signals in navigation and positioning scenarios where conventional satellite navigation is difficult to use [20]. Alongside these navigation-oriented studies, Schneider et al. processed VLF amplitude measurements to deduce quiet-time seasonal variation, showing that VLF amplitude and field-strength observations remain important for analyzing lower-ionospheric propagation effects [21]. These studies demonstrate the continuing value of VLF propagation research. However, many recent works focus on navigation signal detection, PNT system architecture, positioning applications, or amplitude and field-strength variation, while receiver-side timing correction based on phase variation has received relatively less attention. Since positioning, navigation, and timing are closely coupled in PNT applications, improving VLF timing correction can also provide useful support for VLF navigation. Moreover, although field-strength analysis is an important part of propagation theory, phase variation is more directly related to propagation delay. Therefore, this study focuses on delay correction from the perspective of the VLF phase-variation model.

Although VLF propagation modeling and engineering correction methods have been studied for systems such as Omega and Alpha, several issues remain insufficiently addressed for receiver-side timing applications. Existing correction routes often rely on precomputed correction tables, monitoring-station data, or differential correction information. When external correction information is unavailable, propagation modeling provides a feasible way to predict propagation delay under limited observational conditions and to improve the timing accuracy of VLF receiver-side observations. Building on the authors’ previous work on the VLF phase-variation model, this study applies the model to receiver-side delay correction for Alpha/VLF timing observations by estimating the model-predicted delay from propagation path parameters and phase-variation relationships, and then removing it from the observed delay sequence.

This study aims to develop and evaluate a propagation-delay correction method for Alpha/VLF timing observations based on the phase-variation model. The main contributions are as follows. First, the VLF propagation-delay error is decomposed into primary path delay, model-predicted delay, and residual random error, which clarifies the correction object of the study. Second, the phase-velocity expression derived from the Earth–ionosphere waveguide model is used to construct the model-predicted delay, and the correction is applied to receiver-side observed delay sequences. Third, historical Alpha observations from the Chongqing and Guilin receiving sites are used to evaluate the correction performance, and the standard deviation, root mean square error, and relative reduction are used as quantitative metrics. The remainder of this paper is organized as follows. Section 2 presents the theoretical model and correction procedure. Section 3 reports the correction results for the selected Alpha propagation paths. Section 4 discusses the physical interpretation, data characteristics, applicability, and limitations of the proposed method.

## 2. Materials and Methods

Data processing, numerical calculation, and figure plotting were performed using MATLAB R2023b (MathWorks, Natick, MA, USA).

### 2.1. Overview of the Correction Method

This study proposes a propagation-delay correction method for very-low-frequency (VLF) timing signals. During long-distance propagation, VLF signals are mainly affected by the Earth–ionosphere waveguide. The received phase and propagation delay vary with propagation path, effective ionospheric reflection height, day–night variation, and local propagation disturbances. For timing applications, propagation-delay errors directly affect receiver-side time synchronization, so the stable or quasi-periodic components of these errors need to be modeled and corrected.

The receiver-side delay error of a VLF timing signal can be divided into three components: primary path delay, secondary propagation delay, and residual random error. The primary path delay is mainly determined by the propagation distance and propagation velocity between the transmitting station and the receiving site. For a fixed transmitting station and a fixed receiving site, this component is relatively stable and can be calculated from their geographic coordinates. The secondary propagation delay is mainly caused by time-varying propagation conditions, with the day–night variation in the effective ionospheric reflection height being a typical factor. The residual random error includes receiver noise, system-delay uncertainty, local interference, short-term environmental fluctuation, and other effects that are not explicitly modeled.

Based on this decomposition, the correction procedure consists of four main steps. First, the propagation path length is calculated from the geographic coordinates of the transmitting station and the receiving site, and the primary path delay is then obtained from the propagation velocity. Second, the relationship between phase velocity and effective ionospheric reflection height is established using waveguide-mode propagation theory. Third, the model-predicted delay associated with the day–night variation in the effective reflection height is estimated using the phase-variation model. Finally, the model-predicted delay and a constant receiver-related offset are removed from the observed delay sequence to obtain the corrected residual sequence. The correction performance is then evaluated using the standard deviation, root mean square error, and relative reduction.

### 2.2. Theoretical Basis of VLF Earth–Ionosphere Waveguide Propagation

#### 2.2.1. Earth–Ionosphere Waveguide Propagation Characteristics

VLF signals generally occupy the 3–30 kHz frequency band, corresponding to wavelengths of approximately 100–10 km. Because of their long wavelengths, long-distance VLF signals cannot be described simply as conventional shortwave skywave hops. Their propagation is more appropriately treated as propagation in the Earth–ionosphere waveguide, where the ground acts as the lower boundary and the lower ionosphere acts as the upper boundary. The electromagnetic wave is repeatedly reflected between these two boundaries and travels over long distances along the Earth’s surface.

At shorter propagation distances, the received signal may contain ground-wave components, skywave components, and multimode interference. As the propagation distance increases, higher-order modes attenuate more rapidly, and the received signal gradually becomes dominated by low-order modes. Under long-distance propagation conditions, waveguide-mode theory can therefore be used to describe the phase velocity, phase variation, and propagation-delay variation in VLF signals. Since this study focuses on delay correction for long-distance VLF timing signals, the phase-velocity relation under the dominant-mode condition is used as the basis for modeling phase-delay variation.

According to waveguide-mode propagation theory, the propagation phase in the waveguide is related not only to signal frequency and wavelength, but also to the effective ionospheric reflection height, Earth curvature, and propagation mode. In an ideal planar waveguide, the modal condition can be described by a phase-resonance relation after boundary reflection. For longer propagation paths, Earth curvature must also be considered, and the waveguide is better represented by a spherical or approximately spherical model. These relations provide the basis for connecting phase velocity, phase variation, and propagation delay.

#### 2.2.2. Modal Relation and Phase-Velocity Expression

Solving the waveguide-mode equation gives the modal propagation condition for VLF signals in the Earth–ionosphere waveguide and links the modal parameter, effective ionospheric reflection height, and phase velocity [22]. The complete derivation involves waveguide boundary conditions, modal eigenvalues, and electromagnetic-field expressions. Only the relations directly used in the delay-correction model are retained here; the full derivation is given in Appendix A.

In waveguide-mode propagation, stable propagation is associated with resonant modes that satisfy the boundary phase condition. For an equivalent waveguide formed by the ground and the ionosphere, the modal condition can be expressed through the relation between propagation angle, signal wavelength, and effective reflection height. Let n denote the mode order, λ the signal wavelength, and h the effective ionospheric reflection height. The angular parameter of the n-th mode can be written as:

(1) $$C_{n}=\left(n-\frac{1}{2}\right)\frac{\lambda }{2h}$$

Here, $C_{n}$ is determined by the mode order, signal wavelength, and effective reflection height. For a given wavelength, a change in h directly changes the propagation angle in the waveguide and therefore affects the phase velocity.

The phase velocity is the velocity at which the signal phase advances along the propagation direction. From the modal relation, the phase velocity can be expressed as:

(2) $$v_{p}=c{\left(1-C_{n}^{2}\right)}^{-\frac{1}{2}}$$

where $v_{p}$ is the phase velocity without Earth-curvature correction, and c is the speed of light in vacuum. This expression shows that the phase velocity is not a fixed constant. It depends on the modal parameter $C_{n}$, which is further determined by λ, h, and n. A change in effective reflection height, therefore, changes the VLF phase velocity through the modal condition.

For long-distance propagation, the effect of the Earth’s curvature cannot be ignored. With curvature correction included, the phase velocity is written as:

(3) $$v_{p}^{\prime }=c{\left(1-C_{n}^{2}\right)}^{-\frac{1}{2}}\left(1-\frac{h}{2a}\right)$$

where $v_{p}^{\prime }$ is the phase velocity after Earth-curvature correction, and a is the Earth’s radius. Compared with the planar-waveguide expression, the correction factor $\left(1-\frac{h}{2a}\right)$ accounts for the effect of the Earth’s curvature under spherical propagation conditions.

Under long-distance propagation conditions, higher-order modes attenuate more rapidly than the first-order mode, and the received signal is mainly associated with the low-order or first-order dominant mode. For n=1,

(4) $$C_{1}=\frac{\lambda }{4h}$$

The phase velocity of the first-order dominant mode with Earth-curvature correction is therefore:

(5) $$v_{p}^{\prime }=c{\left(1-\frac{\lambda^{2}}{16h^{2}}\right)}^{-\frac{1}{2}}\left(1-\frac{h}{2a}\right)$$

This expression retains the main effects of signal wavelength, Earth radius, and effective ionospheric reflection height on phase velocity. For the same VLF transmitting signal, λ is fixed. For a given Earth model, a is also known. The effective ionospheric reflection height h therefore becomes the key variable controlling the day–night change in phase velocity. This relation is used later to estimate the secondary propagation-delay variation caused by day–night ionospheric changes.

#### 2.2.3. Model Applicability

This study represents long-distance VLF propagation using an equivalent Earth–ionosphere waveguide. Along the selected propagation paths, the received signal is treated mainly as a low-order or dominant-mode signal, and phase-delay variation is described through changes in phase velocity. This treatment is suitable for analyzing periodic phase variation over long-distance paths, especially the delay variation caused by the day–night change in effective ionospheric reflection height.

In the model, the ionospheric state is represented by an effective reflection height. Daytime and nighttime are associated with relatively stable effective reflection heights, while the day–night transition appears as a continuous change in phase and propagation delay. In this way, complex ionospheric parameters such as electron density, collision frequency, and conductivity gradient are represented through the effective reflection height and its variation, so that the delay correction can be formulated using the phase-velocity model.

The model does not explicitly describe all local propagation factors. Sea–land conductivity differences, terrain complexity, local receiver conditions, short-term ionospheric disturbances, strong solar activity, and receiver-delay drift may still affect the corrected residuals. The correction in this study is therefore aimed mainly at the propagation-delay component with a day–night periodic pattern, while unmodeled propagation disturbances and receiver-side random errors remain in the residual sequence.

### 2.3. Delay-Error Composition of VLF Timing Signals

For a VLF timing signal, the receiver-side delay error is caused by the combined effects of the propagation path, propagation environment, transmitting and receiving systems, and random disturbances. To clarify the component corrected in this study, the total receiver-side delay error can be decomposed as:

(6) $$\Delta \tau (t)=\tau_{p}+\tau_{s}(t)+\varepsilon (t)$$

where Δτ(t) is the total receiver-side delay error, $\tau_{p}$ is the primary path delay, $\tau_{s}(t)$ is the time-varying secondary propagation delay, and ε(t) is the residual random error.

The primary path delay $\tau_{p}$ is the stable delay component generated by signal propagation along the transmitter–receiver path. It is mainly determined by the distance between the transmitting station and the receiving site, as well as by the propagation velocity along the path. For a fixed transmitting station and a fixed receiving site, this component remains nearly constant over a short time interval and can be calculated from geographic coordinates and propagation velocity. The primary path delay is usually the largest component of the total propagation delay.

The secondary propagation delay $\tau_{s}(t)$ is caused by time-varying propagation conditions, including changes in the ionospheric state, ground parameters, and path environment. This study focuses on the periodic component caused by the day–night variation in the effective ionospheric reflection height. This component usually shows a clear diurnal pattern, especially during sunrise and sunset transitions. Since this variation has a partly predictable structure, it can be estimated and corrected using the phase-variation model.

The residual random error ε(t) includes the error sources that are not described by the primary path-delay calculation or by the phase-variation model. These sources may include transmitting-system delay uncertainty, receiver thermal noise, environmental noise, short-term propagation fluctuation, local electromagnetic interference, path-medium perturbation, and data-processing error. These errors are generally not deterministic and are therefore not directly predicted by the waveguide-based phase-variation model. In this study, they remain in the corrected residual and are evaluated using statistical metrics.

With this decomposition, the objective of the delay-correction method can be stated more specifically. The stable primary path delay is first calculated from the propagation path, and the day–night periodic component of the secondary propagation delay is then estimated using the phase-variation model. The remaining error after correction is treated as the residual sequence, which is used to evaluate how effectively the main periodic propagation-delay component was suppressed.

### 2.4. Primary Path-Delay Calculation

The primary path delay is the stable delay component determined by the propagation path. For a fixed transmitting station and receiving site, this delay is mainly controlled by the path length and the propagation velocity. In VLF timing applications, the primary path delay must be calculated before the time-varying secondary delay is corrected; otherwise, the fixed delay caused by propagation distance cannot be separated from the delay variation caused by the propagation environment.

Let v denote the propagation velocity of the VLF signal in the atmosphere and d the propagation path length between the transmitting station and the receiving site. The primary path delay is then:

(7) $$\tau_{p}=\frac{d}{v}$$

Considering the effect of the atmospheric refractive index n, the propagation velocity can be written as:

(8) $$v=\frac{c}{n}$$

Therefore, the primary path delay can also be expressed as:

(9) $$\tau_{p}=\frac{nd}{c}$$

Here, c is the speed of light in vacuum, n is the atmospheric refractive index, and d is the propagation path length from the transmitting station to the receiving site. In this study, c is taken as 299,792,458 m/s, and the average surface atmospheric refractive index used in the model is 1.000338.

The path length d is calculated from the geographic coordinates of the transmitting station and the receiving site. For long-distance propagation, a planar-distance approximation is insufficient, and a great-circle or ellipsoidal geodesic distance should be used. The path length can be written as:

(10) $$d=S+\Delta S_{h}$$

where S is the ellipsoidal or great-circle path distance determined from the longitude and latitude of the two sites, and $\Delta S_{h}$ is the distance correction introduced by elevation.

For VLF propagation paths of several thousand kilometers, Earth curvature and reference-ellipsoid parameters affect the path-distance calculation. The Andoyer–Lambert method can be used to calculate an approximate geodesic distance from the spherical distance and ellipsoidal correction, while the Bessel method solves the geodetic problem by projecting the ellipsoidal elements onto an auxiliary sphere and using successive approximation. In this study, the path-distance calculation is based on the coordinates of the transmitting station and the receiving site, with height correction included when necessary. The detailed geodetic calculation and height-correction procedure are given in Appendix B.

In the delay-correction procedure, the primary path-delay calculation provides the stable path-delay reference. This delay is mainly determined by the propagation path and does not describe the day–night variation in the propagation environment. After the primary path delay is calculated, the correction focuses on the time-varying secondary propagation delay.

### 2.5. Phase-Variation-Based Secondary Propagation-Delay Correction

The secondary propagation delay is mainly caused by time-varying propagation conditions. Among these factors, the day–night variation in the effective ionospheric reflection height has a direct influence on the phase delay of VLF signals. During the daytime, solar radiation increases ionization in the lower ionosphere, and the effective reflection height is relatively low. At night, the ionospheric state changes, and the effective reflection height increases [23]. This height variation changes the waveguide propagation condition and leads to variations in both phase velocity and phase delay.

For the dominant mode under long-distance propagation conditions, the phase velocity with Earth-curvature correction can be written as *Equation (5)*, where $v_{p}^{\prime }$ is the phase velocity after Earth-curvature correction, c is the speed of light in vacuum, λ is the signal wavelength, h is the effective ionospheric reflection height, and a is the Earth’s radius. This expression shows that a change in h changes the phase velocity of the VLF signal in the waveguide.

Since the daytime and nighttime effective reflection heights are different, the phase velocities along the same propagation path are also different. Let $v_{d}$ and $v_{n}$ denote the daytime and nighttime phase velocities, respectively, and let d denote the propagation path length. The day–night propagation-delay difference caused by the phase-velocity difference can be expressed as:

(11) $$\Delta \tau_{dn}=d\left(\frac{1}{v_{n}}-\frac{1}{v_{d}}\right)$$

In phase form, the corresponding day–night phase difference is:

(12) $$\Delta \phi_{dn}=2\pi fd\left(\frac{1}{v_{n}}-\frac{1}{v_{d}}\right)$$

where f is the signal frequency, $\Delta \tau_{dn}$ is the day–night propagation-delay difference, and $\Delta \phi_{dn}$ is the day–night phase difference. These equations show that the accumulated phase and delay differences are related to both path length and the difference between daytime and nighttime phase velocities. For the same phase-velocity difference, a longer propagation path produces a larger accumulated delay difference.

Let $h_{d}$ and $h_{n}$ denote the daytime and nighttime effective ionospheric reflection heights, respectively. The corresponding $v_{d}$ and $v_{n}$ can be calculated from the phase-velocity expression above. A larger difference between $h_{d}$ and $h_{n}$ produces a larger difference between daytime and nighttime phase velocities, and therefore a larger secondary propagation-delay variation. The effective reflection heights used in the model are treated as model parameters rather than universal constants. Their values depend on the propagation path, season, ionospheric state, and observation conditions.

Based on the phase-variation model, a model-predicted delay curve can be constructed. The curve describes three intervals: a relatively stable daytime interval, a day–night transition interval, and a relatively stable nighttime interval. During stable daytime and nighttime conditions, the predicted delay changes slowly. During sunrise and sunset transitions, the predicted delay changes more rapidly as the effective reflection height varies along the propagation path. This predicted curve is used as the model-predicted delay for correcting the observed delay sequence.

Let $\tau_{obs}(t)$ be the observed delay sequence and ${\hat{\tau }}_{m}(t)$ be the model-predicted delay. The correction is performed by subtracting ${\hat{\tau }}_{m}(t)$, together with a constant receiver-related offset, from the observed sequence. This operation suppresses the main day–night periodic component in the observed delay sequence. The remaining sequence is then treated as the corrected residual, which contains receiver noise, local propagation disturbances, and other unmodeled effects.

### 2.6. Correction Workflow and Evaluation Metrics

The propagation-delay correction is implemented after the primary path delay and the periodic secondary propagation delay have been determined. The procedure first uses the geographic coordinates of the transmitting station and the receiving site to calculate the path length and the corresponding primary path delay. The phase-variation model is then used to estimate the model-predicted delay associated with the day–night change in effective ionospheric reflection height. The predicted secondary delay is removed from the observed delay sequence together with a constant receiver-related offset, and the remaining sequence is used as the corrected residual.

Let $\tau_{obs}\left(t_{i}\right)$ denote the observed delay sequence at time $t_{i}$, ${\hat{\tau }}_{m}(t_{i})$ the predicted periodic secondary propagation delay, and $\tau_{0}$ the constant receiver-related offset. The corrected residual can be defined as:

(13) $$e(t_{i})=\tau_{obs}(t_{i})-\tau_{0}-{\hat{\tau }}_{m}(t_{i})$$

where $e(t_{i})$ represents the residual delay after correction. The constant term $\tau_{0}$ accounts for the fixed phase or delay bias associated with the receiving system and the initial reference of the observation sequence.

The standard deviation is used to describe the dispersion of the residual sequence:

(14) $$\sigma =\sqrt{\frac{1}{N-1}\sum_{i=1}^{N}(e_{i}-\bar{e}{)}^{2}}$$

where N is the number of samples, $e_{i}$ is the residual delay at the i-th sample, and $\bar{e}$ is the mean residual delay. A smaller standard deviation indicates weaker fluctuation in the corrected residual sequence.

The root mean square error is used to describe the overall deviation of the residual sequence from zero:

(15) $$RMSE=\sqrt{\frac{1}{N}\sum_{i=1}^{N}e_{i}^{2}}$$

Compared with the standard deviation, RMSE is affected by both the fluctuation and the mean offset of the residual sequence, and therefore provides an overall measure of the residual delay level.

To quantify the relative improvement after correction, the relative reduction in a statistical metric is defined as:

(16) $$\eta =\frac{M_{before}-M_{after}}{M_{before}}\times 100\%$$

where $M_{before}$ and $M_{after}$ are the values of the selected metric before and after correction, respectively. In this study, M can represent either the standard deviation or the RMSE. A larger η indicates a greater reduction in the corresponding error metric after correction.

The following section applies these metrics to the selected Alpha observation data. The correction results are reported separately for the Chongqing and Guilin receiving sites, and the relative reductions in the standard deviation and RMSE are calculated to evaluate the suppression of the periodic propagation-delay component.

### 2.7. Comparison with Existing VLF Propagation-Delay Correction Methods

Existing VLF navigation and timing systems usually correct propagation delay by combining propagation modeling, monitored phase data, and user-position information. In the Omega system, propagation correction tables were used to correct received phase measurements. These correction tables were not purely analytical results independent of observations. Instead, they were based on theoretical propagation predictions and were further adjusted using phase data monitored at transmitting stations and other sites. The theoretical model used for the correction tables was revised periodically to account for solar activity and other propagation anomalies, and the propagation correction tables were updated on average every two years [24].

The Omega correction tables also had a grid-based structure. In VLF timing applications, the skywave correction table provided correction values according to transmitting station, frequency, date, time, and receiving region. For temperate regions, the corrections could be tabulated on latitude–longitude grids, and the correction at a receiving site could be obtained by interpolation from the surrounding grid points [25]. Available VLF timing data indicate that, because the published skywave corrections were already sufficiently dense, interpolation from the surrounding grid squares usually improved the prediction by no more than about 5 μs compared with directly using the nominal table value for the grid square containing the receiver. A typical long-term calibration error for either day or night was about 3.25 μs [26].

Differential Omega followed a real-time differential correction approach. A monitor station at a known fixed position observed the local propagation error and provided correction information to users within the differential service area. Public Omega system data report that the conventional Omega mode could provide approximately 1–2 nautical miles (nmi) RMS accuracy when propagation correction tables were used, whereas the Differential or Relative Omega mode could reach approximately 0.1–0.3 nmi RMS [24,27]. This indicates that real-time monitored propagation corrections can further reduce local propagation-related navigation errors.

Unlike the table-based and monitoring-station-based correction routes discussed above, the method in this study estimates the model-predicted delay from the transmitter–receiver path parameters and the phase-variation model. This delay is derived from the phase-velocity variation associated with changes in the effective ionospheric reflection height and is then removed from the receiver-side observed delay sequence. Since some existing Omega references report navigation positioning accuracy, whereas this study evaluates the statistical residual of a corrected delay sequence, these metrics are not directly interchangeable. The comparison here is therefore used to clarify the technical route of the proposed method rather than to make a direct accuracy equivalence. The corrected residual may still contain receiver noise, local propagation disturbances, ground-parameter variation, short-term ionospheric perturbations, and other unmodeled effects.

## 3. Results

Because the Russian Alpha navigation system has remained out of service for unknown reasons over the past two years, this study uses historical measured data instead. The data were taken from the paper “Observation and Analysis of VLF Signal Phase and Field Strength during the Solar Eclipse of 22 July 2009,” published in the *Journal of Time and Frequency* in June 2011. That paper reports measurements collected over five days, from 20 to 25 July 2009, for signals transmitted from the master, eastern, and western stations to Chongqing, Qingdao, and Guilin. In the present study, four datasets recorded on 20 July were selected, corresponding to the paths from the master station and the eastern secondary station to Chongqing and Guilin [28].

### 3.1. Alpha Test Results: Chongqing

The receiving stations were the eastern secondary station, western secondary station, and master station of the Alpha navigation system, and the reception site was Chongqing, China. The data were recorded on 20 July 2009, with each dataset covering one full day. The reception coordinates were $29.5630^{\circ }\;N,106.5516^{\circ }\;E$. The phase variations in the received signals and their corresponding correction results are shown in the Figure below.

**Figure 1:**
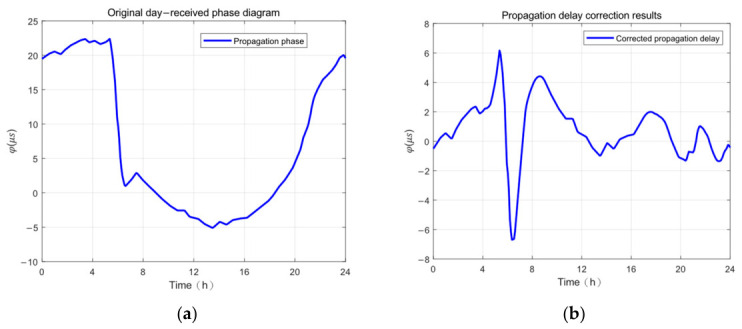
Daily phase variation in the Alpha master-station signal received in Chongqing, China: (**a**) Phase variation before correction; and (**b**) phase variation after correction.

**Figure 2:**
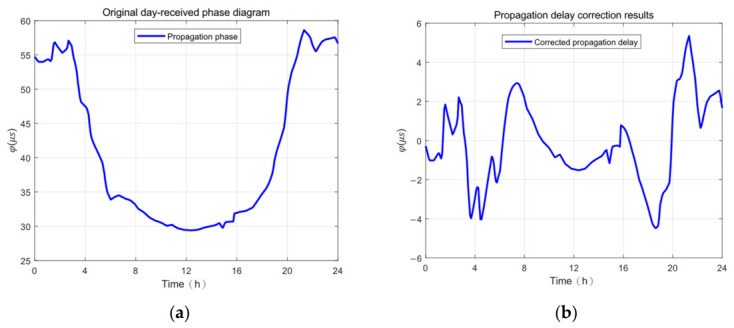
Daily phase variation in the Alpha eastern-station signal received in Chongqing, China: (**a**) Phase variation before correction; and (**b**) phase variation after correction.

**Figure 3:**
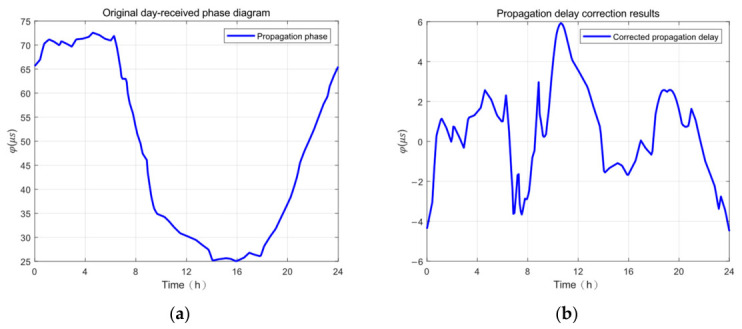
Daily phase variation in the Alpha western-station signal received in Chongqing, China: (**a**) Phase variation before correction; and (**b**) phase variation after correction.

As shown in the pre-correction curves in Figure 1, Figure 2 and Figure 3 and in Table 1, the received Alpha phase over the Chongqing paths exhibits a clear 24 h periodic pattern. The delay remains relatively stable during daytime and nighttime intervals, while the main variation appears during the transition between the two states. This behavior is consistent with the day–night change in the effective ionospheric reflection height described in Section 2.

After propagation-delay correction, the fluctuation of all three Chongqing paths is significantly reduced. As shown in Table 2, the corrected standard deviations decrease to 2.0054–2.2500 μs, and the corrected RMSE values decrease to 2.1316–2.3020 μs. The relative-reduction results in Table 3 further show that the standard deviation decreases by 80.62–87.58%, while the RMSE decreases by 82.60–95.45%. The largest reduction is observed for the western secondary-station path, where the standard deviation decreases from 18.1112 μs to 2.2500 μs and the RMSE decreases from 50.5574 μs to 2.3020 μs.

Although the great-circle distance of the western secondary-station path is much longer than those of the master-station and eastern secondary-station paths, its corrected standard deviation remains close to the values obtained for the other two paths. This indicates that the proposed correction method can suppress the dominant periodic delay variation along the selected Chongqing paths. The remaining residuals are at the few-microsecond level and may still contain receiver noise, local propagation disturbances, and other unmodeled effects.

### 3.2. Alpha Test Results: Guilin

The receiving stations were the eastern secondary station, western secondary station, and master station of the Alpha navigation system, and the reception site was Guilin, China. The data were recorded on 20 July 2009, with each dataset covering one full day. The reception coordinates were $25.274^{\circ }\;N,110.2979^{\circ }\;E$. The phase variations in the received signals and their corresponding correction results are shown in the Figure below.

**Figure 4:**
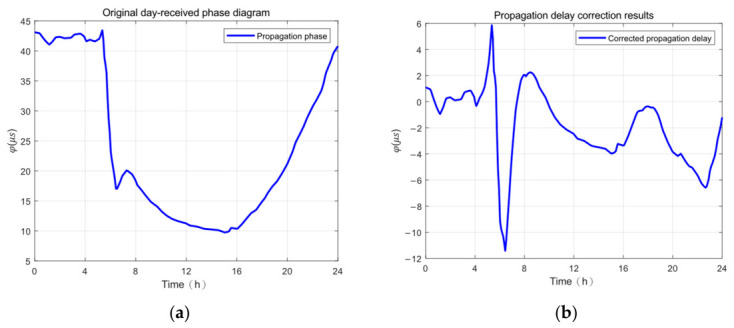
Daily phase variation in the Alpha master-station signal received in Guilin, China: (**a**) Phase variation before correction; and (**b**) phase variation after correction.

**Figure 5:**
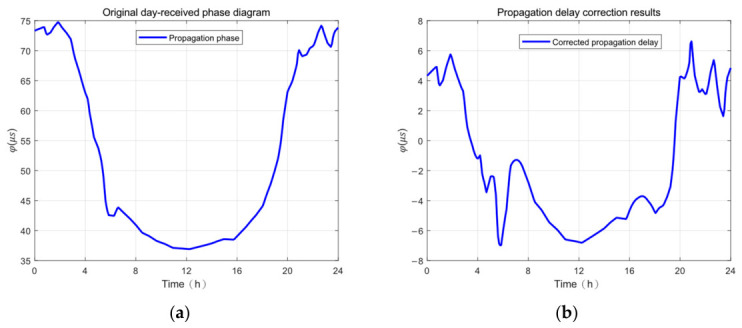
Daily phase variation in the Alpha eastern-station signal received in Guilin, China: (**a**) Phase variation before correction; and (**b**) phase variation after correction.

**Figure 6:**
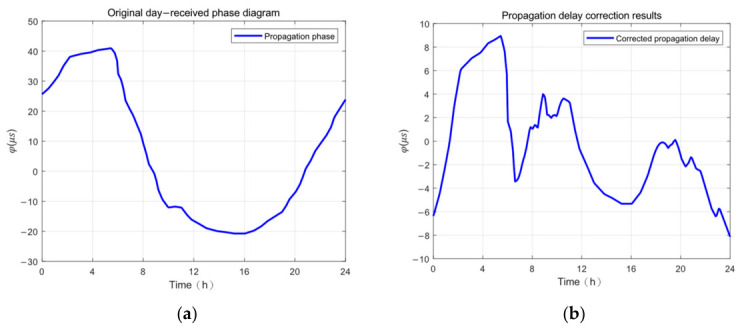
Daily phase variation in the Alpha western-station signal received in Guilin, China: (**a**) Phase variation before correction; and (**b**) phase variation after correction.

As shown in Figure 4, Figure 5 and Figure 6 and Table 4, the Guilin paths also show a clear day–night phase variation. The predicted day–night delay differences are 34.1305 μs for the master-station path, 36.9636 μs for the eastern secondary-station path, and 57.2525 μs for the western secondary-station path. The measured maximum delay differences are close to these predicted values, which indicates that the phase-variation model captures the main periodic delay variation along the selected Guilin paths.

After correction, the standard deviations of the three Guilin paths decrease to 2.7987–4.4792 μs, and the RMSE values decrease to 3.3675–4.5641 μs, as shown in Table 5. The relative reductions in Table 6 show that the standard deviation decreases by 70.87–80.09%, while the RMSE decreases by 80.55–91.56%. Among the three paths, the western secondary-station path has the largest standard deviation reduction, decreasing from 22.4953 μs to 4.4792 μs. The eastern secondary-station path has the largest RMSE reduction, decreasing from 54.0856 μs to 4.5641 μs.

Compared with the Chongqing paths, the corrected residuals for Guilin are slightly larger. This difference may be related to path geometry, local propagation conditions, receiver environment, or unmodeled variations in ground and ionospheric parameters. Even so, the corrected standard deviations and RMSE values remain within the few-microsecond range. These results indicate that the correction method can suppress the dominant periodic delay variation for the selected Guilin paths, although the residual level varies between receiving sites and propagation paths.

### 3.3. Analysis of Experimental Results

As summarized in Table 7, the correction method reduces the delay fluctuation for all six selected Alpha propagation paths. For the Chongqing paths, the corrected standard deviation is 2.0054–2.2500 μs, and the corrected RMSE is 2.1316–2.3020 μs. For the Guilin paths, the corrected standard deviation is 2.7987–4.4792 μs, and the corrected RMSE is 3.3675–4.5641 μs. Across all selected paths, the corrected residuals remain within the few-microsecond range.

The relative-reduction results further indicate that the standard deviation decreases by 70.87–87.58%, and the RMSE decreases by 80.55–95.45% across the six paths. This reduction shows that the phase-variation-based correction method effectively suppresses the dominant periodic delay variation in the observed delay sequences. The improvement is especially evident in the RMSE results, which suggests that the correction reduces both the fluctuation and the large systematic variation associated with the day–night propagation effect.

The corrected residuals for the Guilin paths are slightly larger than those for the Chongqing paths. This difference may be associated with differences in propagation geometry, local receiving environment, ground conductivity, and unmodeled ionospheric variability along the paths. The result indicates that the model can capture the main periodic propagation-delay component, but the residual level still depends on the receiving site and the specific propagation path.

Overall, the results demonstrate that the proposed correction method is suitable for reducing the periodic propagation delay variation in the selected historical Alpha observations. Since the validation is based on historical data and does not include an independent UTC-traceable absolute delay reference, the reported residuals should be interpreted as corrected receiver-side delay residuals rather than the absolute timing accuracy of a complete VLF timing system.

## 4. Discussion

### 4.1. Summary of Main Findings

This study develops a propagation-delay correction method for Alpha/VLF timing observations based on the VLF signal propagation model. The method uses the transmitter–receiver path parameters and the effective ionospheric reflection-height variation to estimate the model-predicted delay, and then removes this delay from the receiver-side observed delay sequence. The purpose of the correction is to suppress the dominant periodic delay variation in the observed Alpha records.

The experimental results for the selected historical Alpha observations show that the correction reduces the delay fluctuation for all six propagation paths. For the Chongqing paths, the corrected standard deviation is 2.0054–2.2500 μs, and the corrected RMSE is 2.1316–2.3020 μs. For the Guilin paths, the corrected standard deviation is 2.7987–4.4792 μs, and the corrected RMSE is 3.3675–4.5641 μs. Across all selected paths, the corrected residuals remain within the few-microsecond range.

The relative-reduction results further show that the standard deviation decreases by 70.87–87.58%, and the RMSE decreases by 80.55–95.45%. These results indicate that the VLF signal propagation model-based correction method can effectively reduce the main periodic delay variation in the selected Alpha observation sequences. Since the historical dataset does not provide an independent UTC-traceable absolute delay reference, the reported values should be interpreted as corrected receiver-side residuals rather than the absolute timing accuracy of a complete VLF timing receiver.

### 4.2. Interpretation of the Correction Results

The results in Section 3 show that the main fluctuation in the Alpha observation delay sequences is closely related to the periodic delay variation associated with day–night phase changes. Before correction, the received delay curves show obvious variations during the transition between daytime and nighttime conditions, while the delay variation remains relatively small during stable daytime and nighttime intervals. This behavior is consistent with the propagation characteristics of VLF signals in the Earth–ionosphere waveguide, where the day–night variation in the effective ionospheric reflection height changes the waveguide propagation condition and, consequently, affects the phase velocity and propagation delay.

After the model-predicted delay is removed, the residual fluctuation is substantially reduced for all selected propagation paths. This indicates that the VLF signal propagation model-based correction method can capture the main deterministic component of the observed delay variation. In other words, the correction effect does not come from directly filtering random noise, but from modeling and removing the systematic phase-delay variation caused by day–night propagation changes.

The simultaneous reduction in standard deviation and RMSE further supports this interpretation. The standard deviation mainly reflects the dispersion of the corrected residual sequence, whereas RMSE is affected by both residual fluctuation and mean offset. The decrease in both metrics indicates that the proposed method reduces not only short-term fluctuation in the observed sequence, but also the larger systematic variation associated with the day–night propagation effect. Therefore, the corrected residuals are closer to a combination of receiver noise, local propagation disturbances, and other unmodeled errors.

### 4.3. Influence of Propagation Path and Receiving Site

The corrected residuals are not identical for the Chongqing and Guilin receiving sites. The Chongqing paths show corrected standard deviations and RMSE values close to approximately 2 μs, whereas the Guilin paths show slightly larger residuals. This difference suggests that the correction performance is influenced by propagation-path characteristics and receiving-site conditions, even when the same VLF signal propagation model is used.

Several factors may contribute to this difference. First, different transmitter–receiver geometries lead to different accumulated phase variations along the propagation path. A longer path can accumulate a larger equivalent delay variation under the same phase-velocity difference. Second, ground conductivity, terrain conditions, and local receiving environments may affect the received phase and introduce residual variations that are not explicitly included in the model. These factors can remain in the corrected residual sequence.

The nonuniformity of the ionosphere may also affect the correction performance. In this study, the ionospheric state is represented by the effective reflection height. This treatment reduces model complexity and can describe the main day–night variation trend. However, the actual ionosphere can be affected by solar activity, geomagnetic conditions, seasonal variation, and local disturbances. Its behavior may therefore deviate from an idealized smooth day–night transition. As a result, the model can suppress the main periodic delay variation, but it cannot completely remove all path-dependent and environment-dependent residuals.

### 4.4. Historical Alpha Data Characteristics and Validation Scope

The validation in this study is based on historical Alpha observations recorded during the July 2009 solar-eclipse observation period. Although the dataset was collected near a total solar eclipse event, the sequences used in this study were recorded on 20 July 2009, whereas the total solar eclipse occurred on 22 July 2009. Therefore, the data used here does not correspond to the eclipse day itself. In the available records, the eclipse day is associated with a distinct propagation-phase disturbance, while the phase variations on the neighboring non-eclipse days exhibit a strong diurnal periodic pattern. The phase values at the same time of day are also relatively close across these non-eclipse days. This suggests that the solar-eclipse effect can be regarded mainly as an abrupt disturbance to VLF propagation, whereas the non-eclipse-day records still retain the regular day–night propagation characteristics needed for model evaluation.

For this reason, the 20 July 2009 records have practical value for evaluating the proposed correction method. They provide complete one-day Alpha phase observations for the selected propagation paths and allow the VLF signal propagation model to be tested against regular day–night phase variation under the available historical data conditions. The use of these data is also meaningful because public Alpha observations with complete path information are limited, and such historical records provide one of the few available bases for examining model-predicted delay correction in long-distance VLF propagation.

Nevertheless, the possible influence of the solar eclipse period cannot be completely excluded. The ionosphere may experience abnormal activity before or after the eclipse day, and such activity may affect VLF propagation in ways that are not fully represented by the regular propagation model. Therefore, the results obtained from the 20 July 2009 records should be interpreted as validation under the available historical Alpha observation conditions, rather than as a general conclusion for all seasons, all Alpha paths, or all ionospheric states.

Another important characteristic of the historical dataset is that it does not provide an independent absolute delay reference traceable to Coordinated Universal Time (UTC). Therefore, the residuals reported in this study should be interpreted as receiver-side corrected residuals after removing the model-predicted delay, rather than as the absolute timing accuracy of a complete VLF timing receiver. Further validation using calibrated receiver-delay information and UTC-traceable timing references would be required to evaluate the absolute timing performance of the method.

### 4.5. Applicability and Future Improvement

The proposed method is most suitable for scenarios in which the main propagation-delay variation is dominated by regular day–night phase changes and no real-time external differential correction is available. Under these conditions, receiver-side continuous phase or delay observations can be combined with path parameters and the VLF signal propagation model to estimate and remove the model-predicted delay. This makes the method useful for historical VLF data analysis, Alpha signal propagation studies, and VLF timing correction under limited monitoring conditions.

Compared with engineering correction routes that depend on propagation-measurement stations or real-time differential corrections, the proposed method does not require an external real-time correction link. Instead, it generates the correction quantity from the physical propagation model. This gives the method practical value when regional monitoring-station data or real-time differential information are unavailable. However, the correction performance depends on the VLF signal propagation model, path-distance calculation, effective ionospheric reflection-height parameters, and the quality of the receiver-side observations. When the propagation environment experiences strong disturbances or the path conditions deviate significantly from the model assumptions, the correction performance may decrease.

Future work can improve the method in three aspects. First, more receiving sites and longer observation periods should be introduced to test the applicability of the method under different seasons, solar-activity levels, and propagation paths. Second, path-dependent ground conductivity, sea–land distribution, and terrain parameters can be incorporated to improve the representation of path differences. Third, calibrated receiver-delay information and UTC-traceable timing references can be introduced so that the corrected results can be further used for evaluating absolute timing performance.

Overall, the results indicate that the VLF signal propagation model-based correction method can reduce the main periodic delay variation in the selected Alpha/VLF observation sequences without relying on real-time external differential corrections. With more complete observation data, path-dependent parameters, and calibrated timing references, the method may be further improved for VLF timing and propagation-delay modeling applications.

## Figures and Tables

**Table 1:** Phase variation in the signal received in Chongqing.

Transmitting Station	Great-Circle Distance	Predicted Daytime Delay	Predicted Nighttime Delay	Predicted Day–Night Delay Difference	Maximum Delay Difference
Master	3395.008 km	11,378.4 μs	11,407.4935 μs	29.0935 μs	27.3880 μs
Eastern	3392.466 km	11,385.5 μs	11,409.5852 μs	24.0852 μs	29.2461 μs
Western	6095.267 km	20,428.4 μs	20,480.6334 μs	52.2334 μs	47.2574 μs

**Table 2:** Comparison of performance metrics before and after propagation-delay correction in Chongqing.

Transmitting Station	Standard Deviation Before Correction	Root Mean Square Error Before Correction	Standard Deviation After Correction	Root Mean Square Error After Correction
Master	10.3489 μs	12.3777 μs	2.0054 μs	2.1537 μs
Eastern	10.9292 μs	42.2693 μs	2.0297 μs	2.1316 μs
Western	18.1112 μs	50.5574 μs	2.2500 μs	2.3020 μs

**Table 3:** Relative reduction in performance metrics after propagation-delay correction in Chongqing.

Transmitting Station	Reduction in Standard Deviation	Reduction in RMSE
Master	80.62%	82.60%
Eastern	81.43%	94.96%
Western	87.58%	95.45%

**Table 4:** Phase variation in the signal received in Guilin.

Transmitting Station	Great-Circle Distance	Predicted Daytime Delay	Predicted Nighttime Delay	Predicted Day–Night Delay Difference	Maximum Delay Difference
Master	3982.786 km	13,348.4 μs	13,382.5305 μs	34.1305 μs	33.6587 μs
Eastern	3562.756 km	11,933.0 μs	11,969.9636 μs	36.9636 μs	37.6818 μs
Western	6680.961 km	22,391.3 μs	22,448.5525 μs	57.2525 μs	61.4393 μs

**Table 5:** Comparison of performance metrics before and after propagation-delay correction in Guilin.

Transmitting Station	Standard Deviation Before Correction	Root Mean Square Error Before Correction	Standard Deviation After Correction	Root Mean Square Error After Correction
Master	12.5630 μs	26.8341 μs	2.7987 μs	3.3675 μs
Eastern	14.5892 μs	54.0856 μs	4.2497 μs	4.5641 μs
Western	22.4953 μs	23.0436 μs	4.4792 μs	4.4826 μs

**Table 6:** Relative reduction in performance metrics after propagation-delay correction in Guilin.

Transmitting Station	Reduction in Standard Deviation	Reduction in RMSE
Master	77.72%	87.45%
Eastern	70.87%	91.56%
Western	80.09%	80.55%

**Table 7:** Summary of correction performance for all selected Alpha propagation paths.

Receiving Site	Corrected Standard Deviation	Corrected RMSE	Reduction in Standard Deviation	Reduction in RMSE
Chongqing	2.0054–2.2500 μs	2.1316–2.3020 μs	80.62–87.58%	82.60–95.45%
Guilin	2.7987–4.4792 μs	3.3675–4.5641 μs	70.87–80.09%	80.55–91.56%
All selected paths	2.0054–4.4792 μs	2.1316–4.5641 μs	70.87–87.58%	80.55–95.45%

## Data Availability

The data used in this study come from the published paper “Observation and Analysis of VLF Signal Phase and Field Strength during the 22 July 2009 Solar Eclipse” by Zhang Shitian, Chen Linru, and Wang Yuanxin, published in the June 2011 issue of the *Journal of Time and Frequency*, pages 66–76, DOI 10.13875/j.issn.1674-0637.2011.01.010 [28]. The original data are publicly available at https://sxtt.cbpt.cnki.net/portal (20 October 2024). We have utilized these data in accordance with the terms and conditions specified by the original authors and the journal.

## Abbreviations

The following abbreviations are used in this manuscript:

PNT	Positioning navigation timing
VLF	Very-low frequency

## Appendix A

The derivation in this appendix follows the framework of classical VLF Earth–ionosphere waveguide-mode theory and is included to preserve the continuity of the calculation procedure.

First, assume the source is a vertical electric dipole located on a smooth spherical Earth surface with radius * a *, conductivity *σ*, and dielectric constant *ε*. The spherical coordinates are (r, θ, ϕ). The dipole is chosen to be located at *r* = *a* and *θ* = 0. For harmonic time dependence, the radial electric field components are written in the following form:

(A1) $$E_{r}≅\frac{e^{-ika\theta }}{a(\theta sin\;\theta {)}^{1/2}}V_{0}$$

Apart from one constant factor, in the case of an airless Earth, the ionosphere is neglected, and $V_{0}$ can be written in the following form:

(A2) $$V_{0}≅2(\pi x{)}^{1/2}e^{-i\pi /4}\sum_{s=1,2,\ldots .}^{\infty }\frac{e^{-ixt_{s}}}{\left(t_{s}-q^{2}\right)}\frac{w_{1}(t_{s}-y)}{w_{1}(t_{s})}$$

where

(A3) $$x={\left(\frac{ka}{2}\right)}^{1/3}\theta$$

(A4) $$y={\left(\frac{2}{ka}\right)}^{1/3}k\left(r-a\right)$$

(A5) $$iq={\left(\frac{ka}{2}\right)}^{1/3}\Delta$$

(A6) $$\Delta ={\left(\frac{i\varepsilon_{0}\omega }{\sigma +i\varepsilon \omega }\right)}^{1/2}{\left(1-\frac{i\varepsilon_{0}\omega }{\sigma +i\varepsilon \omega }\right)}^{1/2}$$

The coefficient $t_{s}$ is a solution to the following equation:

(A7) $$w_{1}^{\prime }(t)-qw_{1}(t)=0$$

where $w_{1}(t)$ is the Airy integral, and the apostrophe indicates the derivative with respect to t. For the third-order Hankel function, we have:

(A8) $$w_{1}(t)=exp(-2\pi i/3)(-\pi t/3{)}^{\frac{1}{2}}H_{\frac{1}{3}}^{\left(2\right)}[(2/3)(-t{)}^{3/2}]$$

The formula for $V_{0}$ is commonly known as the residue-series representation of the ground-wave field. It applies under the conditions ka≫1 and r−a≪a. In addition, the dominant modes must satisfy $|t_{s}|\approx 1$, or at least should not deviate substantially from unity. If $V_{0}$ is expressed in contour-integral form, an equivalent representation is obtained, provided that the integration contours enclose the pole at $t=t_{s}$ in the clockwise direction:

(A9) $$w_{1}^{\prime \prime }(t_{s})-qw_{1}^{\prime }(t_{s})=(t_{s}-q^{2})w_{1}(t_{s})$$

where

(A10) $$w_{1}^{\prime \prime }\left(t\right)=tw_{1}\left(t\right)\;$$

(A11) $$w_{1}^{\prime }(t_{s})=qw_{1}(t_{s})$$

We now consider the Earth to be enclosed by a concentric reflecting shell at r=a+h. The electrical properties of this shell are left unspecified; it is only assumed that upward-propagating waves are reflected downward. Under this assumption, after a single reflection, we have:

(A12) $$e^{-ixt}w_{1}(t-y)$$

transform the above expression into

(A13) $$A(t)e^{-ixt}w_{2}(t-y)$$

where

(A14) $$w_{2}(t)=exp(2\pi i/3)(-\pi t/3{)}^{\frac{1}{2}}H_{\frac{1}{3}}^{\left(1\right)}[(2/3)(-t{)}^{3/2}]$$

A(t) is an unknown function of t. The boundary conditions at r=a+h are written in the following form:

(A15) $${\left[\frac{dw\left(t-y\right)}{dy}-q_{i}w\left(t-y\right)\right]}_{y=y_{0}}=0$$

where

(A16) $$y_{0}={\left(\frac{2}{ka}\right)}^{1/3}kh$$

(A17) $$iq_{i}={\left(\frac{ka}{2}\right)}^{1/3}\Delta_{i}$$

The parameter $\Delta_{i}$ is determined by the properties of the medium beyond r=a+h, and its explicit form need not be specified at this stage. Formally, $\Delta_{i}=Z/\eta_{0}$, where Z denotes the radial surface impedance at r=a+h. In the most general case, $\Delta_{i}$ (or equivalently Z) depends on t, although it is usually treated as a constant. It then follows that:

(A18) $$A\left(t\right)=-\frac{w_{1}^{\prime }\left(t-y_{0}\right)+q_{i}w_{1}\left(t-y_{0}\right)}{w_{2}^{\prime }\left(t-y_{0}\right)+q_{i}w_{2}\left(t-y_{0}\right)}$$

The downward-propagating wave, represented by the function $w_{2}(t-y)$, is then reflected at the ground and gives rise to a new upward-propagating wave of the following form:

(A19) $$A(t)B(t)w_{1}(t-y)e^{-ixt}$$

The boundary conditions at r=a can be written as:

(A20) $${\left[\frac{dw\left(t-y\right)}{dy}+qw\left(t-y\right)\right]}_{y=0}=0$$

Then, by expressing w(t−y) as the sum of the downward-propagating waves and upward-propagating waves, we obtain:

(A21) $$B\left(t\right)=-\frac{w_{2}^{\prime }\left(t\right)-qw_{2}\left(t\right)}{w_{1}^{\prime }\left(t\right)-qw_{1}\left(t\right)}$$

Because the wave alternates continuously between downward- and upward-propagating states as a result of repeated reflections between the Earth and the ionosphere, the total field may be written as:

(A22) $$E_{r}=\frac{e^{-ika\theta }}{a(\theta sin\;\theta {)}^{1/2}}V$$

where

(A23) $$V=\sum_{j=0,1,2\ldots }V_{j}=-2(\pi x{)}^{1/2}e^{-i\pi /4}\sum_{n=0,1,2\ldots }\frac{e^{-ixt_{n}}[w_{1}(t_{n}-y)+A(t_{n})w_{2}(t_{n}-y)]}{[w_{1}^{\prime }(t_{n})-qw_{1}(t_{n})]{\left[\frac{\partial }{\partial t}A(t)B(t)\right]}_{t=t_{n}}}$$

The above equation is valid under the conditions ka≫1 and h≪a. If (−t)≫1, then

(A24) $$B(t)=-\frac{w_{2}^{\prime }(t)-qw_{2}(t)}{w_{1}^{\prime }(t)-qw_{1}(t)}≅[\frac{-i(-t{)}^{1/2}-q}{i(-t{)}^{1/2}-q}]e^{-i\pi /2}exp[i\frac{4}{3}(-t{)}^{3/2}]$$

The modal equation for the coefficient $t_{n}$, 1−A(t)B(t)=0 may be written in the following approximate form:

(A25) RgRiexp(−iI^)=1=e−i2πn

where

(A26) $$R_{g}=\frac{C-\Delta }{C+\Delta }$$

(A27) $$R_{i}=\frac{C^{\prime }-\Delta_{i}}{C^{\prime }+\Delta_{i}}$$

(A28) I^=2ka3[(C′)3−C3]=2ka3[(C2+2h/a)3/2−C3]

If $h/a\ll C^{2}$, then $R_{i}=(C-\Delta_{i})/(C+\Delta_{i})$ and I^≅2khC, which reduces to the modal equations for the flat-Earth case. For numerical calculations, the most convenient form is:

(A29) $${\left[\frac{\partial }{\partial t}A\left(t\right)B\left(t\right)\right]}_{t=t_{n}}$$

This can be expressed using Airy functions and other relationships as follows:

(A30) $$\begin{array}{cc} {\left[\frac{\partial }{\partial t}A\left(t\right)B\left(t\right)\right]}_{t=t_{n}}= & \frac{2i(t_{n}-q^{2})}{\left[w_{1}^{\prime }(t_{n})-qw_{1}(t_{n})][w_{2}^{\prime }(t_{n})-qw_{2}(t_{n})\right]}\\ & -\frac{2i(t_{n}-y_{0}-q_{i}^{2})[w_{2}^{\prime }(t_{n})-qw_{2}(t_{n})]}{[w_{1}^{\prime }(t_{n})-qw_{1}(t_{n})]{\left[w_{2}^{\prime }(t_{n}-y_{0})+q_{i}w_{2}(t_{n}-y_{0})\right]}^{2}} \end{array}$$

Therefore, the complete remainder series can be written as:

(A31) $$V=(\pi x{)}^{1/2}e^{-i\pi /4}\sum_{n=0,1,2}\frac{e^{-ixt_{n}}[w_{1}(t_{n}-y)+A(t_{n})w_{2}(t_{n}-y)]}{\left[\frac{t_{n}-q^{2}}{w_{2}^{\prime }(t_{n})-qw_{2}(t_{n})}-\frac{\left(t_{n}-y_{0}-q_{i}^{2})[w_{2}^{\prime }(t_{n})-qw_{2}(t_{n})\right]}{{\left[w_{2}^{\prime }(t_{n}-y_{0})+q_{i}w_{2}(t_{n}-y_{0})\right]}^{2}}\right]}$$

The modal equation 1−A(t)B(t)=0, which determines the root $t_{n}$, may be written as:

(A32) $$[\frac{w_{2}^{\prime }(t)-qw_{2}(t)}{w_{1}^{\prime }(t)-qw_{1}(t)}][\frac{w_{1}^{\prime }(t-y_{0})+q_{i}w_{1}(t-y_{0})}{w_{2}^{\prime }(t-y_{0})+q_{i}w_{2}(t-y_{0})}]=e^{-i2\pi n}$$

This formula can be simplified to:

(A33) $$R_{g}R_{i}F_{g}e^{-it}=e^{-i2\pi n}$$

where

(A34) I^=23ka[(C′)3−C3]

(A35) $$R_{g}=\frac{C-\Delta }{C+\Delta }$$

(A36) $$R_{i}=\frac{C^{\prime }-\Delta_{i}}{C^{\prime }+\Delta_{i}}$$

(A37) $$(-t{)}^{1/2}=(ka/2{)}^{1/3}C$$

(A38) $$C^{\prime }={\left(C^{2}+2h/a\right)}^{1/2}$$

(A39) $$F_{g}=\frac{\frac{w_{2}^{\prime }\left(t\right)-qw_{2}\left(t\right)}{w_{1}^{\prime }\left(t\right)-qw_{1}\left(t\right)}}{iexp\;i\frac{4}{3}(-t{)}^{\frac{3}{2}}\frac{-i(-t{)}^{\frac{1}{2}}-q}{i(-t{)}^{\frac{1}{2}}-q}}$$

Furthermore, when ${\left(ka/2\right)}^{1/3}C\gg 1$, $F_{g}$ may be taken as unity. Under this condition, Equations (A33) and (A25) are equivalent. In many practical applications, however, $F_{g}$ cannot be approximated in this way. The pattern equation may then be written as:

(A40) $$\frac{2}{3}ka{\left(C^{2}+\frac{2h}{a}\right)}^{3/2}+i\alpha_{1}{\left(C^{2}+\frac{2h}{a}\right)}^{1/2}+ilog\left[\frac{w_{2}^{\prime }\left(t\right)-qw_{2}\left(t\right)}{w_{1}^{\prime }\left(t\right)-qw_{1}\left(t\right)}\right]-\left(4n-1\right)\frac{\pi }{2}=0$$

where $\alpha_{1}$ is a function of C, and, when $|C^{\prime }/\Delta_{i}|\ll 1$,

(A41) $$\alpha_{1}≅-\frac{2}{\Delta_{i}}=-2\frac{\eta_{0}}{Z_{i}}$$

To solve the mode equations, we first assume ideal reflecting boundaries. Specifically, the ground is taken to be perfectly conducting, so that q=0 or $R_{g}=1$, while the ionosphere is treated as a perfect reflector, so that $\alpha_{1}=0$ or $R_{i}=-1$. Let $k=(\varepsilon_{0}\mu_{0}{)}^{1/2}\omega =2\pi /\lambda$. Under these assumptions, the mode equations reduce to:

(A42) $${\left(C^{2}+\frac{2h}{a}\right)}^{3/2}=\frac{3}{2ka}\left[\left(4n-1\right)\frac{\pi }{2}+2{tan}^{-1}\left(\frac{v^{\prime }\left(t\right)}{u^{\prime }\left(t\right)}\right)\right]$$

Solving for the real roots of t yields the following relation:

(A43) $$\frac{v}{c}-1=\frac{1}{Re\;S}-1≅\frac{C^{2}}{2}$$

The phase velocity v can be expressed as:

(A44) $$v=c/S_{n}$$

Here, $S_{n}$ denotes sinθ for the n-th mode. Accordingly, v can also be written as:

(A45) $$v=c{\left(1-C_{n}^{2}\right)}^{-1/2}$$

For long-range propagation, when the effect of the Earth’s curvature is taken into account, the phase velocity is expressed as:

(A46) $$v^{\prime }=v\left(1-\frac{h}{2a}\right)=c{\left(1-C_{n}^{2}\right)}^{-1/2}\left(1-\frac{h}{2a}\right)$$

Under long-range propagation conditions, higher-order modes attenuate much more rapidly than the first-order mode. As a result, only the first-order mode remains observable, and the phase velocity can be written as:

(A47) $$v^{\prime }=c{\left(1-\frac{\lambda^{2}}{16h^{2}}\right)}^{-1/2}\left(1-\frac{h}{2a}\right)$$

## Appendix B

The geodetic-distance calculation in this appendix follows standard ellipsoidal geodesy. The Andoyer–Lambert formula is used as an approximate long-line geodesic-distance method based on ellipsoidal correction terms, while the Bessel method and related geodesic algorithms provide the basis for solving geodetic direct and inverse problems on the reference ellipsoid [27,29,30,31,32,33,34]. Height correction is included because the actual propagation path may differ from the reference-ellipsoid surface when the transmitting and receiving sites have non-negligible elevations [33].

1. Andoyer–Lambert method

The great-circle distance is calculated from the spherical distance on the reference ellipsoid. The distance between two points on the ellipsoidal surface is computed using the Andoyer–Lambert formula [29,30]. Let a and b denote the semi-major and semi-minor axes of the ellipsoid, respectively, and let (L, B) represent the geodetic longitude and latitude of a point on the ellipsoidal surface. Then, the relationship between the spherical coordinates (λ, ϕ) and the geodetic coordinates can be written as [30,31]:

(A48) $$\left\{\begin{array}{c} \mathrm{tg}\varphi =\frac{b}{a}\mathrm{tg}\;B\\ \lambda =L \end{array}\right.$$

Given two points on the ellipsoidal surface with longitude and latitude coordinates $\left(\lambda ,\phi \right)$ and $\left(\lambda_{1},\phi_{1}\right)$, respectively, the distance between them can be expressed as:

(A49) $$S=\delta_{0}+\delta_{s}$$

(A50) $$cos\;\delta_{0}=sin\;\varphi sin\;\varphi_{1}+cos\;\varphi cos\;\varphi_{i}cos\;\left(\lambda -\lambda_{1}\right)$$

(A51) $$\delta_{s}=\frac{f}{4}\left[\frac{3sin\;\delta_{0}-\delta_{0}}{1+cos\;\delta_{0}}{\left(sin\;\varphi +sin\;\varphi_{1}\right)}^{2}-\frac{3sin\;\delta_{0}+\delta_{0}}{1-cos\;\delta_{0}}{\left(sin\;\varphi -sin\;\varphi_{1}\right)}^{2}\right]$$

where a and b are the semi-major and semi-minor axes of the reference ellipsoid, respectively; f is the flattening of the ellipsoid, defined as f=(a−b)/a; and $\delta_{s}$ is the correction from the spherical distance between the two points to the corresponding distance on the ellipsoidal surface. The spherical distance can be calculated as:

(A52) $$d=Rarccos\;\left[sin\;\varphi_{1}sin\;\varphi +cos\;\varphi_{1}cos\;\varphi cos\;\left(\lambda_{1}-\lambda \right)\right]$$

2. Bessel method

The basic idea of the Bessel method for solving the geodetic direct problem is to project the geodetic elements on the ellipsoidal surface onto an auxiliary sphere in accordance with the Bessel projection conditions [32,33,34]. The geodetic direct problem is then solved on the spherical surface, and the results are subsequently transformed back to the ellipsoidal surface [25,26,27,28]. The procedure for determining the geodesic distance S between the start point $\left(L_{1},B_{1}\right)$ and the end point $\left(L_{2},B_{2}\right)$ is given below [27,32,33,34]:

(A53) $$W_{1}=\sqrt{1-e^{2}{sin}^{2}\;B_{1}}$$

(A54) $$W_{2}=\sqrt{1-e^{2}{sin}^{2}\;B_{2}}$$

(A55) $$sin\;u_{1}=\frac{sin\;B_{1}\sqrt{1-e^{2}}}{W_{1}}$$

(A56) $$sin\;u_{2}=\frac{sin\;B_{1}\sqrt{1-e^{2}}}{W_{2}}$$

(A57) $$cos\;u_{1}=\frac{\cos\;B_{1}}{W_{1}}$$

(A58) $$cos\;u_{2}=\frac{\cos\;B_{2}}{W_{2}}$$

(A59) $$L=L_{2}-L_{1}$$

(A60) $$a_{1}=sin\;u_{1}sin\;u_{2}$$

(A61) $$a_{2}=cos\;u_{1}cos\;u_{2}$$

(A62) $$b_{1}=cos\;u_{1}sin\;u_{2}$$

(A63) $$b_{2}=sin\;u_{1}cos\;u_{2}$$

The geodetic azimuth $A_{1}$, the spherical arc length x, and the longitude difference λ=L+δ are computed simultaneously by successive approximation, with δ=0 taken in the first iteration:

(A64) $$p=\cos\;u_{2}\sin\;\lambda$$

(A65) $$q=b_{1}-b_{2}\cos\;\lambda$$

(A66) $$A_{1}=arctan\;\frac{p}{q}$$

**Table A1:** Values of p and q.

Signs of p	Signs of q	$A_{1}$
+	+	$\left|A_{1}\right|$
+	-	$180{}^{\circ}-\left|A_{1}\right|$
-	-	$180{}^{\circ}+\left|A_{1}\right|$
-	+	$360{}^{\circ}-\left|A_{1}\right|$

(A67)
$$\sin\;\sigma =\mathrm{psin}\;A_{1}+\mathrm{qsin}\;A$$

(A68)
$$\cos\;\sigma =a_{1}+a_{2}\cos\;\lambda$$

(A69)
$$\sigma =arctan\;\frac{\sin\;\sigma }{\cos\;\sigma }$$

**Table A2:** Values of cosσ.

Signs of cos σ	σ
+	|σ|
-	180°−|σ|

$\left|A_{1}\right|$ and |σ| are angles in the first quadrant.

(A70) $$sin\;A_{0}=cos\;u_{1}sin\;A_{1}$$

(A71) $$x=2a_{1}-{cos}^{2}\;A_{0}cos\;\sigma$$

(A72) $$\delta =\left[\alpha \sigma -\beta^{\prime }xsin\;\sigma \right]sin\;A_{0}$$

(A73) $$\alpha =\left[33523299-\left(28189-70{cos}^{2}\;A_{0}\right){cos}^{2}\;A_{0}\right]\times 10^{-10}$$

(A74) $$\beta^{\prime }=\left(28189-94{cos}^{2}\;A_{0}\right)\times 10^{-10}$$

Using the computed δ, one obtains λ=L+δ. The updated λ is then used to recalculate δ following the same procedure. This iteration continues until the values of δ in the last two steps are identical or differ by less than a prescribed tolerance. The final values of λ, $A_{1}$, σ, x, and $\sin A_{0}$ are then used in the subsequent calculations:

(A75) $$A=6356863.020+\left(10708.949-13.474\cos^{2}\;A_{0}\right)\cos^{2}\;A_{0}$$

(A76) $$B^{\prime \prime }=\frac{2B}{\cos^{2}\;A_{0}}=10708.938-17.956\cos^{2}\;A_{0}$$

(A77) $$C^{\prime \prime }=\frac{2C}{\cos^{4}\;A_{0}}=4.487$$

(A78) $$y=\left({cos}^{4}\;A_{0}-2x^{2}\right)cos\;\sigma$$

(A79) $$S=A\sigma +\left(B^{\prime \prime }x+C^{\prime \prime }y\right)sin\;\sigma$$

Compute the back azimuth:

(A80) $$A_{2}=arctg\;\left(\frac{cos\;u_{1}sin\;\lambda }{b_{1}cos\;\lambda -b_{2}}\right)$$

The sign of $A_{2}$ is determined in the same manner as that of $A_{1}$. To ensure that the computed great-circle distance D is accurate to within 30 m, the geodetic longitude and latitude of both the transmitting station and the user location must first be determined to within 0.5 arcseconds. The Earth model adopted here is the Krasovsky rotational ellipsoid, as listed in the Table A3 below:

**Table A3:** Values of Earth-related parameters under different modes.

Parameters	Krasovsky Ellipsoid	WGS-84	CGCS2000
a	6,378,245 (m)	6,378,137 (m)	6,378,137 (m)
b	6,356,863.0187730473 (m)	6,356,752.3142 (m)	6,356,725.314 (m)
c	6,399,698.9017827110 (m)	6,399,593.6258 (m)	6,399,593.6259 (m)
α	1/298.3	1/298.257223563	1/298.257222
$e^{2}$	0.006693421622966	0.00669437999013	0.00669438002290
$e^{\prime 2}$	0.006738525414683	0.00673949674227	0.00673949677548

When the distance between two points is large and the elevation (the sum of orthometric height and height anomaly) is also considerable, the effect of elevation must be taken into account; otherwise, the required computational accuracy cannot be achieved. A height correction must therefore be introduced. The formula for the elevation correction to the distance is given by:

(A81) $$\begin{array}{cc} \Delta d= & H[\left(cos\left(B_{2}\right)cos\left(L_{2}\right)-cos\left(B_{1}\right)cos\left(L_{1}\right)\right)\cdot \Delta X\\ & +\left(cos\left(B_{2}\right)sin\left(L_{2}\right)-cos\left(B_{1}\right)sin\left(L_{1}\right)\right)\cdot \Delta Y\\ & +\left(sin\left(B_{2}\right)-sin\left(B_{1}\right)\right)\cdot \Delta Z]/d_{0} \end{array}$$

where H is the mean elevation of the transmitting and receiving points, with elevation defined as the sum of orthometric height and height anomaly:

(A82) $$\Delta X=X_{1}-X_{2}$$

(A83) $$\Delta Y=Y_{1}-Y_{2}$$

(A84) $$\Delta Z=Z_{1}-Z_{2}$$

(A85) $$X_{i}=N_{i}\cos\left(B_{i}\right)\cos\left(L_{i}\right)\;\;\;(i=1,2)$$

(A86) $$Y_{i}=N_{i}\cos\left(B_{i}\right)\sin\left(L_{i}\right)\;\;(i=1,2)$$

(A87) $$Z_{i}=N_{i}\left(1-e^{2}\right)\sin\left(B_{i}\right)\;\;\;(i=1,2)$$

(A88) $$Ni=a/\sqrt{1-e^{2}\sin^{2}\left(B_{i}\right)\;\;\;}(i=1,2)$$

## References

[1] DARPA STOIC: Spatial, Temporal, and Orientation Information in Contested Environments. https://www.darpa.mil/research/programs/spatial-temporal-orientation-information-contested-environments.

[2] Mason R., Bonomo J., Conley T., Consaul R., Frelinger D.R. (2021). Analyzing a More Resilient National Positioning, Navigation, and Timing Capability.

[3] Radoš K., Brkić M., Begušić D. (2024). Recent Advances on Jamming and Spoofing Detection in GNSS. Sensors.

[4] Bowhill S.A. (1961). Diversity effects in long distance high frequency radio pulse propagation. J. Res. Natl. Bur. Stand. D Radio Propag..

[5] Indira Devi M., Khan I., Madhusudhana Rao D.N. (2008). A study of VLF wave propagation characteristics in the Earth-ionosphere waveguide. Earth Planets Space.

[6] Wait J.R., Spies K.P. (1964). Characteristics of the Earth-Ionosphere Waveguide for VLF Radio Waves.

[7] Jin Z.W. (2008). Propagation of VLF/SLF waves and their application in submarine communication and navigation. Equip. Environ. Eng..

[8] Burns S., Gasdia F., Simpson J.J., Marshall R.A. (2021). 3-D FDTD Modeling of Long-Distance VLF Propagation in the Earth-Ionosphere Waveguide. IEEE Trans. Antennas Propag..

[9] Barr R., Jones D.L., Rodger C.J. (2000). ELF and VLF radio waves. J. Atmos. Sol. Terr. Phys..

[10] Maley S.W., Bahar E. (1964). Effects of wall perturbations in multimode waveguides. Radio Sci. J. Res. NBS/USNC URS.

[11] Xu W., Gu X., Ni B., Wang S., Yang Z., Cheng W., Hu Z.-J., He F., Li B., Chen X.-C. (2023). Measurements and Modeling of the Responses of VLF Transmitter Signals to X-Class Solar Flares at the Great Wall Station in Antarctica. Space Weather.

[12] Gu X., Chen H., Wang S., Lu Z., Ni B., Li G., Cheng W. (2022). Extraction of Alpha Transmitter Signals from Single-Station Observations Using the Direction-Finding Method. Sci. China Technol. Sci..

[13] Norton K.A. (1953). Transmission loss in radio propagation. Proc. IRE.

[14] Belcher S.R.G., Clilverd M.A., Rodger C.J., Cook S., Thomson N.R., Brundell J.B., Raita T. (2021). Solar Flare X-Ray Impacts on Long Subionospheric VLF Paths. Space Weather.

[15] Morgan A.H., Baltzer O.J. (1964). A VLF Timing Experiment. Radio Sci. J. Res. NBS/USNC-URSI.

[16] Lawrence R.S., Jespersen J.L., Lamb R.C. (1961). Digital methods for the extraction of phase and amplitude information from a modulated signal. J. Res. Natl. Bur. Stand. D Radio Propag..

[17] Creamer P.M., Gupta R.R., Morris P.B. (1985). OMEGA Navigation System Position-Fix Accuracy Assessment. Navigation.

[18] Yan W., Li S., Ma X., Song Y., Yuan J., Hua Y. (2024). Research on an Alpha Navigation Signal Detection Method Based on Multichannel Orthogonal Correlation. Appl. Sci..

[19] Yang Y., Ren X., Jia X., Sun B. (2023). Development Trends of the National Secure PNT System Based on BDS. Sci. China Earth Sci..

[20] Sakaya S., Takahashi M. (2025). Investigation of VLF Electromagnetic Undersea Positioning System Using Receiving Antenna Array in a Simulation. IEEE Access.

[21] Schneider H., Wendt V., Banys D., Clilverd M., Raita T. (2024). Processing of VLF Amplitude Measurements: Deduction of a Quiet Time Seasonal Variation. Radio Sci..

[22] Wait J.R. (1962). An Analysis of VLF Mode Propagation for a Variable Ionosphere Height. J. Res. Natl. Bur. Stand. D Radio Propag..

[23] Gu T.T., Xu H.L., Li K. (2018). Mode interferences of VLF waves in an anisotropic waveguide due to sunrise and sunset. IEEE Trans. Antennas Propag..

[24] Asche G.P. (1973). The Omega System of Global Navigation. Int. Hydrogr. Rev..

[25] Chi A.R., Fletcher L.A., Casselman C.J. (1971). Omega Time Transmissions and Receiving Requirements.

[26] Swanson E.R. (1972). Use of Propagation Corrections for VLF Timing. Proceedings of the Fourth Precise Time and Time Interval Planning Meeting.

[27] Goodman G.R. (1969). A Proposed Differential Omega System. Master’s Thesis.

[28] Zhang S., Chen L., Wang Y. (2011). Observation and analysis of VLF phase/amplitude during a solar eclipse of July 22, 2009. J. Time Freq..

[29] Thomas P.D. (1965). Geodesic Arc Length on the Reference Ellipsoid to Second-Order Terms in the Flattening. J. Geophys. Res..

[30] Vincenty T. (1975). Direct and Inverse Solutions of Geodesics on the Ellipsoid with Application of Nested Equations. Surv. Rev..

[31] Jekeli C. (2016). Geometric Reference Systems in Geodesy.

[32] Bessel F.W. (2010). The Calculation of Longitude and Latitude from Geodesic Measurements (1825). Astron. Nachr..

[33] Rapp R.H. (1993). Geometric Geodesy, Part II.

[34] Karney C.F.F. (2013). Algorithms for Geodesics. J. Geod..

